# Dyspraxia and autistic traits in adults with and without autism spectrum conditions

**DOI:** 10.1186/s13229-016-0112-x

**Published:** 2016-11-25

**Authors:** Sarah Cassidy, Penelope Hannant, Teresa Tavassoli, Carrie Allison, Paula Smith, Simon Baron-Cohen

**Affiliations:** 1 Centre for Psychology, Behaviour and Achievement, Coventry University, Priory Street, Coventry, CV1 5FB UK; 2 Autism Research Centre, Department of Psychiatry, University of Cambridge, Douglas House, 18B Trumpington Road, Cambridge, CB2 8AH UK; 3 The Centre for Research in Autism and Education, UCL Institute of Education, University College London, 55-59 Gordon Square, London, WC1H 0NU UK; 4 CLASS Clinic, Cambridgeshire and Peterborough NHS Foundation Trust, Cambridge, UK

**Keywords:** Autism spectrum conditions, Dyspraxia, Co-morbidity, Autistic traits, Social skills

## Abstract

**Background:**

Autism spectrum conditions (ASC) are frequently associated with motor coordination difficulties. However, no studies have explored the prevalence of dyspraxia in a large sample of individuals with and without ASC or associations between dyspraxia and autistic traits in these individuals.

**Methods:**

Two thousand eight hundred seventy-one adults (with ASC) and 10,706 controls (without ASC) self-reported whether they have been diagnosed with dyspraxia. A subsample of participants then completed the Autism Spectrum Quotient (AQ; 1237 ASC and 6765 controls) and the Empathy Quotient (EQ; 1147 ASC and 6129 controls) online through the Autism Research Centre website. The prevalence of dyspraxia was compared between those with and without ASC. AQ and EQ scores were compared across the four groups: (1) adults with ASC with dyspraxia, (2) adults with ASC without dyspraxia, (3) controls with dyspraxia, and (4) controls without dyspraxia.

**Results:**

Adults with ASC were significantly more likely to report a diagnosis of dyspraxia (6.9%) than those without ASC (0.8%). In the ASC group, those with co-morbid diagnosis of dyspraxia did not have significantly different AQ or EQ scores than those without co-morbid dyspraxia. However, in the control group (without ASC), those with dyspraxia had significantly higher AQ and lower EQ scores than those without dyspraxia.

**Conclusions:**

Dyspraxia is significantly more prevalent in adults with ASC compared to controls, confirming reports that motor coordination difficulties are significantly more common in this group. Interestingly, in the general population, dyspraxia was associated with significantly higher autistic traits and lower empathy. These results suggest that motor coordination skills are important for effective social skills and empathy.

## Background

Is ability to effectively coordinate, plan and carry out movements associated with successful social functioning? Dyspraxia is characterized by pronounced difficulties in the selection, timing and spatial organization of purposeful movement and coordination [1] and is thought to arise from atypical neural connections in the cerebral cortex [2]. Individuals with autism spectrum conditions (ASC), who have pronounced difficulties with social interaction, also exhibit atypical motor movements [3]. In fact, original clinical reports of ASC reported general ‘clumsiness’ in these individuals [4, 5], which have been recently confirmed in a number of research studies [6, 7]. Children (without ASC), who have dyspraxia, also exhibit social and emotional difficulties [8]. However, little research in ASC or the general population has explored the association between dyspraxia and social or emotional skills in adulthood [9]. This is the purpose of the current study.

Studies of children with ASC have demonstrated significant motor difficulties in these individuals [10, 11]. Motor skill scores for children with ASC often fall 1.5 standard deviations below the typical mean [12, 13], and approximately 80% have definite pronounced motor difficulties with 10% being borderline [6, 14–18]. Atypical motor skills in ASC are present from early infancy [19–21] and reported by parents as one of their first areas of concern (average age 14.7 months) prior to seeking an ASC diagnosis [22]. Motor difficulties in children with ASC may be associated with their social and communicative difficulties. For example, children with ASC show significant difficulties in skilled motor gestures, such as imitation [23]. Empathic ability is also reduced in children (without ASC) who exhibit motor difficulties [8], and research has shown correlations between motor coordination and social communication skills in children with ASC [24–26]. Approximately 2–6% of children (without ASC) from the general population have dyspraxia [27]. These children exhibit difficulties in social skills, social phobia, empathy [8, 28], maintaining peer relationships and increased anxiety [29–31]. This suggests that children without ASC, but with dyspraxia, exhibit traits associated with ASC, particularly in social interaction, empathy and social anxiety.

There are however very few studies on dyspraxia in adults with and without ASC—all of the aforementioned studies involved children. However, the limited number of available studies suggests that in those with and without ASC, motor difficulties continue into adulthood [32, 33]. There is also preliminary evidence from a small number of studies that general population young adults (aged 16–25 years) with dyspraxia may have many of the same difficulties as in childhood [34, 35]. There is also high risk of these individuals experiencing mental health problems, low self-esteem and emotional difficulties, exacerbated by low occupational attainment [9]. It is also currently unclear how many adults with and without ASC have dyspraxia or the impact of this on their social skills.

To address this gap in research, the current study aimed to explore the prevalence of dyspraxia in a large population sample of adults with and without ASC and associations between dyspraxia and autistic traits in these individuals. We utilize online self-reported diagnosis of dyspraxia, alongside validated measures of autistic traits (the Autism Spectrum Quotient (AQ) [36]) and empathy (the Empathy Quotient (EQ) [37]). These measures have been validated for use in those with ASC and the general population, to reliably quantify individual differences in autistic traits and empathy in those with and without ASC. This allows us to assess whether the presence of dyspraxia is associated with significantly increased autistic traits in a large population sample, consisting of over 2500 adults with ASC and over 10,000 adults without ASC. Recruitment of high-functioning adults over 18 years old also allows us to explore associations between dyspraxia and autistic traits independent of intellectual disability or age-related effects through development.

If movement difficulties are significantly associated with social, communication skills and empathy, then individuals with ASC and co-morbid dyspraxia may have a significantly higher number of autistic traits than individuals with ASC without co-morbid dyspraxia. Additionally, individuals (without ASC) who have dyspraxia may also have significantly higher autistic traits than those without dyspraxia or ASC. If this were the case, then this would suggest that movement difficulties are associated with autistic traits in those with and without ASC and could be a prime target for intervention to improve social skills in these groups.

## Methods

### Participants

Participants completed questionnaires online through one of two websites (www.autismresearchcentre.com or www.cambridgepsychology.com). Controls (without ASC) were only included if they did not report having a child or other family members with autism, to avoid including those with the ‘broader autism phenotype’ [38]. Individuals with a diagnosis of bipolar disorder, epilepsy, schizophrenia, attention deficit/hyperactivity disorder (ADHD), obsessive-compulsive disorder (OCD), learning disability (LD), intersex/transsexual condition or psychosis were excluded from the control group.

After exclusions, 2871 participants reported having a formal clinical diagnosis of ASC (70% male). A majority (*n* = 2056) had Asperger syndrome; the remaining participants reported having high-functioning autism (*n* = 287), autism (*n* = 302), atypical autism (n=43), pervasive developmental disorder (*n* = 124) and autism spectrum condition (i.e. participants who did not specify a subtype) (*n* = 59). The control group (without ASC) was comprised of *n* = 10,706 individuals (41% male), who reported they had no diagnosis of ASC. Participants were aged between 18 and 75 years old (Table 1).

**Table 1:** Self-reported dyspraxia in adults with ASC vs. adult controls without ASC

	Dyspraxia	No dyspraxia
ASC (*n* = 2871)	199 (6.9%)	2672 (93.1%)
Control (*n* = 10,706)	91 (0.8%)	10,615 (99.2%)
Total (*n* = 13,577)	290 (2.1%)	13,287 (97.9%)

A majority of the individuals in the ASC group provided information on type of education (mainstream, home, special) (*n* = 2473, 86%), and of these individuals, a majority reported having attended mainstream school (*n* = 1949, 78.8%). In total, 1284 (44.7%) of the ASC group also provided information on current occupation, and of these, a majority (*n* = 846, 65.9%) were employed, *n* = 233 (18.1%) individuals were in full-time study and *n* = 202 (15.7%) individuals were unemployed. In the control group, *n* = 5490 (51.3%) individuals provided information on their education type, and of these, a majority (*n* = 5358, 97.6%) reported having attended mainstream education. In total, 6011 (56.1%) of the control group provided information on their occupation, and of these, *n* = 4931 (82%) were currently employed, *n* = 967 (16.1%) were in full-time study and *n* = 113 (1.9%) were unemployed.

### Measures

When registering in the CARD, participants provided demographic data, including age, biological birth sex and educational and occupational attainment, and any diagnoses from a trained clinician, including ASC and dyspraxia. Participants then complete questionnaires designed to quantify autistic traits. We extracted data from two of these self-report questionnaires. The AQ [36] quantifies individual differences in autistic traits, in adults with average or above average intelligence quotient (IQ). The EQ quantifies individual differences in empathizing ability [37].

### Statistical analysis

Chi-square analyses were used to compare the prevalence of dyspraxia in the ASC and control groups, with odds ratios used as a measure of effect size. Large samples increase the robustness of ANOVA to violation of normality and homogeneity of variance. Separate two-way ANCOVAs, including age as a covariate, were conducted on AQ and EQ data, with two between-subjects factors of ‘diagnosis’ (ASC vs. control) and ‘dyspraxia’ (dyspraxia vs. no dyspraxia). The presence of significant diagnosis-by-dyspraxia interaction effects indicates that the effect of dyspraxia on autistic traits is dependent on ASC diagnosis. Significant interaction effects were followed up by simple main effects analysis, to establish whether the effect of dyspraxia on autistic traits was present in each diagnostic group. Effect sizes were calculated using partial eta squared (*η* ^2^) for main effects, interactions and simple main effects. For partial eta squared (*η* ^2^), 0.01 represents a small, 0.06 a medium and 0.14 a large effect.

## Results

Table 1 shows the frequency of self-reported dyspraxia in the ASC and control groups. Table 2 shows the means for age, AQ and EQ for (1) adults with ASC with dyspraxia, (2) adults with ASC without dyspraxia, (3) controls with dyspraxia, and (4) controls without dyspraxia.

**Table 2:** Mean age, AQ and EQ by diagnostic group

	Dyspraxia?	Age (SD)	AQ (SD)	EQ (SD)
ASC	Yes	29 (12.4)	37.6 (7.5)	20 (12)
	No	35.7 (13.2)	38.5 (6.8)	17.6 (9.9)
Control	Yes	25.3 (10.1)	23.4 (8.2)	39.8 (14.4)
	No	31.4 (13.5)	17.7 (7.5)	45 (14.2)
Total	Yes	27.9 (11.9)	33.4 (10)	25.2 (15.3)
	No	32.1 (13.5)	20.7 (10)	41 (16.8)

### Dyspraxia

Participants with ASC were significantly more likely to report a clinical diagnosis of dyspraxia (6.9%) than controls (0.8%) (*X* ^2^(1) = 400.5, *p* < 0.001; OR 8.69).

### AQ

A between-subjects ANCOVA showed a significant main effect of age (*F*(1, 7997) = 131, *p* < 0.001, *η* ^2^ = 0.02). After controlling for the effect of age, there was a significant main effect of dyspraxia, where participants with a diagnosis of dyspraxia self-reported significantly higher levels of autistic traits (mean = 33.4) than those without a diagnosis of dyspraxia (mean = 20.7) (*F*(1, 7997) = 16.58, *p* < 0.001, *η* ^2^ = 0.002). Results also showed a significant main effect of ASC diagnosis, where participants with a diagnosis of ASC self-reported significantly higher levels of autistic traits (mean = 38.4) than those without ASC (mean = 17.8) (*F*(1, 7997) = 624, *p* < 0.001, *η* ^2^ = 0.07). Lastly, there was a significant interaction between the presence of dyspraxia and ASC diagnosis (*F*(1, 7997) = 22, *p* < 0.001, *η* ^2^ = 0.003). Simple main effects analysis showed a significant effect of dyspraxia in the control group (*F*(1, 7997) = 28, *p* < 0.001, *η* ^2^ = 0.003); controls with dyspraxia self-reported significantly higher levels of autistic traits (mean = 23.4) than controls without dyspraxia (mean = 17.7). There was no significant effect of dyspraxia in the ASC group (*F*(1, 7997) = 0.3, *p* = 0.5, *η* ^2^ = 0.001).

### EQ

A between-subjects ANCOVA showed a significant main effect of age (*F*(1, 7271) = 18, *p* < 0.001, *η* ^2^ = 0.002). After controlling for the effect of age, there was no significant main effect of dyspraxia; participants with a diagnosis of dyspraxia did not self-report significantly different levels of empathy (mean = 25.2) than those without a diagnosis of dyspraxia (mean = 41) (*F*(1, 7271) = 0.6, *p* =.4, *η* ^2^ = 0.001). However, results did show a significant main effect of ASC diagnosis, where participants with a diagnosis of ASC self-reported significantly lower empathy (mean = 17.8) than those without ASC (mean = 45) (*F*(1, 7271) = 289, *p* < 0.001, *η* ^2^ = 0.04). Lastly, there was a significant interaction between the presence of dyspraxia and ASC diagnosis (*F*(1, 7271) = 8, *p* < 0.01, *η* ^2^ = 0.001). Simple main effects analysis showed a significant effect of dyspraxia in the control group (*F*(1, 7271) = 4, *p* = 0.04, *η* ^2^ = 0.01); controls with dyspraxia self-reported significantly lower empathy (mean = 39.8) than controls without dyspraxia (mean = 45). There was no significant effect of dyspraxia in the ASC group (*F*(1, 7271) = 3.6, *p* = 0.06, *η* ^2^ = 0.001).

## Discussion

This study aimed to explore for the first time whether dyspraxia was significantly more prevalent in adults with ASC compared to controls without ASC and associations between dyspraxia and autistic traits in adults with and without ASC. Results showed that adults with ASC self-reported a significantly higher rate of dyspraxia (6.9%) than adults without ASC (0.8%); the relative odds of having a diagnosis of dyspraxia were 8 times higher in adults with ASC compared to controls without ASC. These results show for the first time that the prevalence of dyspraxia is significantly higher in adults with ASC compared to controls without ASC. These findings reflect previous research, showing that motor coordination difficulties are highly prevalent in ASC [6, 7]. Furthermore, these findings add to the small body of currently available evidence showing that the difficulties associated with dyspraxia in childhood persist into adulthood [33, 35].

Results also showed that the association between dyspraxia and levels of autistic traits and empathy differed according to the presence of co-morbid ASC. Specifically, diagnosis of dyspraxia was only significantly associated with self-reported autistic traits and empathy if participants did not have co-morbid ASC. Controls without ASC, with a diagnosis of dyspraxia, self-reported a significantly higher number of autistic traits and significantly lower levels of empathy than controls without ASC or dyspraxia, whereas those with ASC and co-morbid dyspraxia did not self-report significantly different levels of autistic traits or empathy compared to those with ASC without co-morbid dyspraxia.

These results suggest that motor coordination difficulties are significantly associated with social skills and empathy in adults without ASC, whereas co-morbid dyspraxia in adults with ASC is not significantly associated with increased difficulties in social communication skills and empathy. One possible explanation for this finding is that dyspraxia and ASC symptoms may overlap, particularly as both conditions are seemingly associated with atypical development of neurons within the cerebral cortex [2, 7, 39]. For example, previous research has shown that the difficulties individuals with dyspraxia experience are somewhat similar to the difficulties people with ASC experience. Empathy, for example, is significantly reduced in children (without ASC) who exhibit motor difficulties [8]. Hence, the association between dyspraxia with social skills and empathy in those without diagnosis of ASC would be greater than in those with co-morbid ASC.

Another possibility is that dyspraxia may be under-diagnosed in those with ASC. Motor difficulties are highly prevalent in people with ASC [6, 7], and these difficulties may be viewed as part of their ASC, as opposed to requiring another co-morbid diagnosis. However, given the small but growing body of evidence showing the importance of motor coordination in social skills in both the general population (without ASC) and those with ASC, recognition and diagnosis of these difficulties is key to access appropriate support and treatment. A small body of evidence suggests high risk of adults with dyspraxia, without co-morbid ASC, experiencing mental health problems, low self-esteem and emotional difficulties, exacerbated by low occupational attainment [9]. Results from the current study add to this literature, suggesting that these individuals also experience difficulties in social skills and empathy, characteristic of ASC. Future research will need to explore whether improving motor coordination early in childhood, or in adulthood, improves these poor outcomes.

The current study has a number of strengths as well as limitations. It contributes to an under-explored area of research—dyspraxia in adulthood and the prevalence and impact of dyspraxia on autistic traits in adults with and without ASC. It also utilized measures of self-reported autistic traits (AQ) and empathy (EQ), which both have undergone substantial reliability tests and have excellent psychometric properties [40, 41]. The current study also analysed data from a very large population sample—over 2800 adults with ASC and 10,000 control adults without ASC. These large numbers were necessary in order to explore differences in autistic traits between the groups, considering that only 0.8% of control adults had a diagnosis of dyspraxia. One limitation is that in order to achieve this large sample, self-report measures of dyspraxia and ASC diagnoses were necessary. However, previous research has shown significantly high concordance rates between self-reported and clinically confirmed diagnoses [42, 43]. Additionally, participants provide details on when and where they received their ASC diagnosis when they register in the research database, to ensure that these self-reported diagnoses are valid. Hence, it is unlikely that the self-report methods utilized in the study significantly invalidated the results.

Another potential limitation is the use of the term ‘dyspraxia’ in the current study. This could have led to an under-reporting of this diagnosis in the control group, due to lack of familiarity with this term. It may also be the case that the likelihood of receiving a dyspraxia diagnosis maybe unevenly distributed across subsets of those with ASC. As discussed above, it is possible that dyspraxia is under-diagnosed in those with ASC, and this may differ by subtype. This could have meant that the rate of dyspraxia in both the control and ASC groups could have been under-estimated in the current study. However, if anything, this means that the rate of dyspraxia diagnosis is a conservative estimate in the current study. If an alternative label, or in person measures, were used, the rates could potentially have been higher in both groups. Future research studies will need to explore whether these rates of dyspraxia are replicable in a large representative sample of those with and without ASC using in person assessments, across the autism spectrum. However, taken together, this is the only and the largest study to date that has explored the prevalence of dyspraxia and associations between dyspraxia and autistic traits in those with and without ASC.

## Conclusions

In conclusion, the current study reports the first evidence that dyspraxia is significantly more prevalent in adults with ASC compared to controls without ASC, confirming previous reports that motor coordination difficulties are highly prevalent in these individuals. Interestingly, the presence of dyspraxia was significantly associated with difficulties in social skills and empathy, particularly in those without co-morbid ASC. These results suggest that adults with dyspraxia demonstrate a significantly increased number of autistic traits compared to the general population (without ASC or dyspraxia) and thus experience similar difficulties to adults with ASC. This is the first evidence of the significant association between motor coordination difficulties with social skills and empathy in adults in the general population and adds to the limited available literature showing a host of poor outcomes in adults with dyspraxia (without ASC). Clinicians must be aware of the impact and importance of motor coordination skills for wider social functioning and empathy and offer appropriate support and treatment for these individuals.

## Abbreviations

AQ: Autism Spectrum Quotient
ASC: Autism spectrum condition
EQ: Empathy Quotient

## References

[1] Grieve J, Gnanasekaran L. Neuropsychology for occupational therapists: cognition in occupational performance. Oxford: Blackwell Publishing; 2008.

[2] Portwood M. Developmental dyspraxia: identification and intervention: a manual for parents and professionals. London: Routledge; 2013.

[3] American Psychiatric Association. Diagnostic and statistical manual of mental disorders (DSM-5®). American Psychiatric Pub; 2013.

[4] Kanner L. Autistic disturbances of affective contact. Publisher not identified; 1943. p. 217-250.

[5] Asperger H (1944). Die “Autistischen Psychopathen” im Kindesalter. Eur Arch Psychiatry Clin Neurosci.

[6] Gowen E, Hamilton A (2013). Motor abilities in autism: a review using a computational context. J Autism Dev Disord.

[7] Hannant P, Cassidy SA, Tavissoli T. The role of sensorimotor difficulties in the development of autism spectrum conditions. Frontiers Neurology: Movement Disorders. 2016; 7. 10.3389/fneur.2016.00124 PMC4978940 27559329

[8] Cummins A, Piek JP, Dyck MJ (2005). Motor coordination, empathy, and social behaviour in school**-**aged children. Dev Med Child Neurol.

[9] Kirby A, Williams N, Thomas M, Hill EL (2013). Self-reported mood, general health, wellbeing and employment status in adults with suspected DCD. Res Dev Disabil.

[10] Berkeley SL, Zittel LL, Pitney LV, Nichols SE (2001). Locomotor and object control skills of children diagnosed with autism. Adapt Phys Act Q.

[11] Hughes C (1996). Brief report: planning problems in autism at the level of motor control. J Autism Dev Disord.

[12] Miyahara M, Tsujii M, Hori M, Nakanishi K, Kageyama H, Sugiyama T (1997). Brief report: motor incoordination in children with Asperger syndrome and learning disabilities. J Autism Dev Disord.

[13] Fournier KA, Hass CJ, Naik SK, Lodha N, Cauraugh JH (2010). Motor coordination in autism spectrum disorders: a synthesis and meta-analysis. J Autism Dev Disord.

[14] Whyatt CP, Craig CM (2012). Motor skills in children aged 7–10 years, diagnosed with autism spectrum disorder. J Autism Dev Disord.

[15] Whyatt C, Craig C (2013). Sensory-motor problems in Autism. Front Integr Neurosci.

[16] Green D, Charman T, Pickles A, Chandler S, Loucas T, Simonoff E, Baird G (2009). Impairment in movement skills of children with autistic spectrum disorders. Dev Med Child Neurol.

[17] Ming X, Brimacombe M, Wagner GC (2007). Prevalence of motor impairment in autism spectrum disorders. Brain Dev.

[18] Page J, Boucher J (1998). Motor impairments in children with autistic disorder. Child Lang Teach Ther.

[19] Teitelbaum P, Teitelbaum O, Nye J, Fryman J, Maurer RG (1998). Movement analysis in infancy may be useful for early diagnosis of autism. Proc Natl Acad Sci.

[20] Matson JL, Mahan S, Fodstad JC, Hess JA, Neal D (2010). Motor skill abilities in toddlers with autistic disorder, pervasive developmental disorder—not otherwise specified, and atypical development. Res Autism Spectr Disord.

[21] Bhat AN, Galloway JC, Landa RJ (2012). Relation between early motor delay and later communication delay in infants at risk for autism. Infant Behav Dev.

[22] Chawarska K, Paul R, Klin A, Hannigen S, Dichtel LE, Volkmar F (2007). Parental recognition of developmental problems in toddlers with autism spectrum disorders. J Autism Dev Disord.

[23] Mostofsky SH, Dubey P, Jerath VK, Jansiewicz EM, Goldberg MC, Denckla MB (2006). Developmental dyspraxia is not limited to imitation in children with autism spectrum disorders. J Int Neuropsychol Soc.

[24] MacDonald M, Lord C, Ulrich DA (2014). Motor skills and calibrated autism severity in young children with autism spectrum disorder. Adapt Phys Act Q.

[25] Dziuk MA, Larson JC, Apostu A, Mahone EM, Denckla MB, Mostofsky SH (2007). Dyspraxia in autism: association with motor, social, and communicative deficits. Dev Med Child Neurol.

[26] Hilton C, Wente L, LaVesser P, Ito M, Reed C, Herzberg G (2007). Relationship between motor skill impairment and severity in children with Asperger syndrome. Res Autism Spectr Disord.

[27] Lingam R, Hunt L, Golding J, Jongmans M, Emond A (2009). Prevalence of developmental coordination disorder using the DSM-IV at 7 years of age: a UK population-based study. Pediatrics.

[28] Pratt ML, Hill EL (2011). Anxiety profiles in children with and without developmental coordination disorder. Res Dev Disabil.

[29] Pearsall-Jones JG, Piek JP, Rigoli D, Martin NC, Levy F (2011). Motor disorder and anxious and depressive symptomatology: a monozygotic co-twin control approach. Res Dev Disabil.

[30] Dewey D, Kaplan BJ, Crawford SG, Wilson BN (2002). Developmental coordination disorder: associated problems in attention, learning, and psychosocial adjustment. Hum Mov Sci.

[31] Smyth MM, Anderson HI (2000). Coping with clumsiness in the school playground: social and physical play in children with coordination impairments. Br J Dev Psychol.

[32] Cantell MH, Smyth MM, Ahonen TP (2003). Two distinct pathways for developmental coordination disorder: persistence and resolution. Hum Mov Sci.

[33] Losse A, Henderson SE, Elliman D, Hall D, Knight E, Jongmans M (1991). Clumsiness in children—do they grow out of it? A 10‐year follow‐up study. Dev Med Child Neurol.

[34] Hill EL, Brown D, Sorgardt KS (2001). A preliminary investigation of quality of life satisfaction reports in emerging adults with and without developmental coordination disorder. J Adult Dev.

[35] Kirby A, Edwards L, Sugden D (2011). Emerging adulthood in developmental co-ordination disorder: parent and young adult perspectives. Res Dev Disabil.

[36] Baron-Cohen S, Wheelwright S, Skinner R, Martin J, Clubley E (2001). The autism-spectrum quotient (AQ): evidence from Asperger syndrome/high-functioning autism, males and females, scientists and mathematicians. J Autism Dev Disord.

[37] Baron-Cohen S, Wheelwright S (2004). The empathy quotient: an investigation of adults with Asperger syndrome or high functioning autism, and normal sex differences. J Autism Dev Disord.

[38] Piven J, Palmer P, Jacobi D, Childress D, Arndt S (1997). Broader autism phenotype: evidence from a family history study of multiple-incidence autism families. Am J Psychiatr.

[39] Fatemi SH, Aldinger KA, Ashwood P, Bauman ML, Blaha CD, Blatt GJ, Chauhan A, Chauhan V, Dager SR, Dickson PE, Estes AM (2012). Consensus paper: pathological role of the cerebellum in autism. Cerebellum.

[40] Ruzich E, Allison C, Smith P, Watson P, Auyeung B, Ring H, Baron-Cohen S (2015). Measuring autistic traits in the general population: a systematic review of the Autism-Spectrum Quotient (AQ) in a nonclinical population sample of 6,900 typical adult males and females. Mol Autism.

[41] Allison C, Baron-Cohen S, Wheelwright SJ, Stone MH, Muncer SJ (2011). Psychometric analysis of the Empathy Quotient (EQ). Personal Individ Differ.

[42] Auyeung B, Allison C, Wheelwright S, Baron-Cohen S (2012). Brief report: development of the adolescent empathy and systemizing quotients. J Autism Dev Disord.

[43] Daniels AM, Rosenberg RE, Anderson C, Law JK, Marvin AR, Law PA (2012). Verification of parent-report of child autism spectrum disorder diagnosis to a web-based autism registry. J Autism Dev Disord.

